# Development of a Single Nucleotide Polymorphism Barcode to Genotype *Plasmodium vivax* Infections

**DOI:** 10.1371/journal.pntd.0003539

**Published:** 2015-03-17

**Authors:** Mary Lynn Baniecki, Aubrey L. Faust, Stephen F. Schaffner, Daniel J. Park, Kevin Galinsky, Rachel F. Daniels, Elizabeth Hamilton, Marcelo U. Ferreira, Nadira D. Karunaweera, David Serre, Peter A. Zimmerman, Juliana M. Sá, Thomas E. Wellems, Lise Musset, Eric Legrand, Alexandre Melnikov, Daniel E. Neafsey, Sarah K. Volkman, Dyann F. Wirth, Pardis C. Sabeti

**Affiliations:** 1 Broad Institute, Cambridge, Massachusetts, United States of America; 2 Department of Organismic and Evolutionary Biology, Harvard University, Cambridge, Massachusetts, United States of America; 3 Department of Immunology and Infectious Diseases, Harvard School of Public Health, Boston, Massachusetts, United States of America; 4 Department of Parasitology, University of São Paulo, São Paulo, Brazil; 5 Department of Parasitology, Faculty of Medicine, University of Colombo, Colombo, Sri Lanka; 6 Department of Genomic Medicine Institute, Cleveland Clinic Lerner Research Institute, Cleveland, Ohio, United States of America; 7 Department of International Health, Biology and Genetics, Case Western Reserve University, Cleveland, Ohio, United States of America; 8 Laboratory of Malaria and Vector Research, Malaria Genetics Section, National Institute of Allergy and Infectious Diseases, Rockville, Maryland, United States of America; 9 Department of Parasitology, Institute Pasteur de la Guyane, Cayenne, French Guiana; 10 School of Nursing and Health Sciences, Simmons College, Boston, Massachusetts, United States of America; Walter and Eliza Hall Institute, AUSTRALIA

## Abstract

*Plasmodium vivax*, one of the five species of *Plasmodium* parasites that cause human malaria, is responsible for 25–40% of malaria cases worldwide. Malaria global elimination efforts will benefit from accurate and effective genotyping tools that will provide insight into the population genetics and diversity of this parasite. The recent sequencing of *P. vivax* isolates from South America, Africa, and Asia presents a new opportunity by uncovering thousands of novel single nucleotide polymorphisms (SNPs). Genotyping a selection of these SNPs provides a robust, low-cost method of identifying parasite infections through their unique genetic signature or barcode. Based on our experience in generating a SNP barcode for *P. falciparum* using High Resolution Melting (HRM), we have developed a similar tool for *P. vivax*. We selected globally polymorphic SNPs from available *P. vivax* genome sequence data that were located in putatively selectively neutral sites (i.e., intergenic, intronic, or 4-fold degenerate coding). From these candidate SNPs we defined a barcode consisting of 42 SNPs. We analyzed the performance of the 42-SNP barcode on 87 *P. vivax* clinical samples from parasite populations in South America (Brazil, French Guiana), Africa (Ethiopia) and Asia (Sri Lanka). We found that the *P. vivax* barcode is robust, as it requires only a small quantity of DNA (limit of detection 0.3 ng/μl) to yield reproducible genotype calls, and detects polymorphic genotypes with high sensitivity. The markers are informative across all clinical samples evaluated (average minor allele frequency > 0.1). Population genetic and statistical analyses show the barcode captures high degrees of population diversity and differentiates geographically distinct populations. Our 42-SNP barcode provides a robust, informative, and standardized genetic marker set that accurately identifies a genomic signature for *P. vivax* infections.

## Author Summary

*Plasmodium vivax* malaria is a major global public health problem, with nearly 2.5 billion people at risk for infection and approximately 132–391 million clinical infections annually. It has a wide geographical range, with a high disease burden in Asia, Central and South America, the Middle East, Oceania, and East Africa. Advances in sequencing technology and sample processing have made it possible to characterize the genetic diversity of *P. vivax* populations. This genetic variation provides a means to identify parasites by unique genetic signatures, or “barcodes.” We developed such a genetic barcode for *P. vivax*, composed of 42 robust and informative variants. Here we report its development and validation based on 87 clinical samples identified by microscopy to contain *P. vivax* from geographically diverse parasite populations from South America (Brazil, French Guiana), Africa (Ethiopia) and Asia (Sri Lanka). We show that the SNP barcode provides a genotyping tool that can be performed at low cost, providing a means to uniquely identify parasite infections and distinguish geographic origins, and that barcode data may offer new insights into *P. vivax* population structure and diversity.

## Introduction

*P. vivax* is a significant disease threat and the most widely distributed human malaria parasite [1]. Its disease burden is imposed predominantly on Asia, Central and South America, the Middle East, Oceania, and East Africa, where nearly 2.5 billion people are at risk for infection [1] and approximately 132–391 million clinical infections are reported annually [2]. Historically, *P. vivax* malaria has been considered relatively benign as it has produced lower mortality than *P. falciparum* [2], the most virulent parasite and the predominant parasite in Africa. This perceived lack of severity, combined with an inability to maintain continuous *in vitro* cell culture, has hampered research on *P. vivax* for decades [3,4]. Nevertheless, reports of drug resistance [5] and increased virulence [6] make *P. vivax* elimination efforts urgent.

Genotyping assays with high sensitivity, simplicity, and low cost are powerful tools to identify parasite infections and may provide insight into the population genetics and diversity of *P. vivax* infections over time [7]. Highly polymorphic microsatellites have been the mainstay of this kind of analysis, revealing high levels of genetic diversity and multiple-clone infections [8–11] and proving useful for mapping focal outbreaks within countries [7,12–14]. However, the interpretation of microsatellite assays can be difficult to standardize across laboratories, and microsatellites are less amenable than SNPs to high-throughput genotyping [15].

The recent whole genome sequencing of numerous *P. vivax* genomes has been an important advance, uncovering a large number of SNPs [16–20] that could potentially be adapted to genotyping applications. Through these sequencing efforts, researchers have discovered that *P. vivax* is nearly twice as diverse as *P. falciparum* at the global population level [20]. This parasite genomic diversity can be explored efficiently in larger collections of samples with a SNP molecular barcode. A SNP barcode contains a combination of SNPs that together express the unique pattern of variation for the parasite sequence. Several successful SNP barcodes have been developed and deployed for *P. falciparum* [21–24] using high resolution melting (HRM) analysis. Their utility has been validated as genotyping tools to monitor malaria transmission, evaluate interventions, and pursue local and regional malaria elimination [25].

HRM is a simple, rapid and low-cost SNP genotyping method based on amplicon melting. Its advantages are that it requires only a saturating double stranded DNA dye and a post-PCR short melting step [26]. The SNP allele is identified by the amplicon melting curve, which depends on its sequence length, percent GC content, and heterozygosity. By melting the amplicon products of a PCR reaction through a gradual increase in temperature, slight genetic differences such as SNPs can be detected by monitoring the dissociation rate of the DNA with a dye and plotting the progress as a melt curve [27]. This approach provides a robust, cost-efficient, closed-tubed system that allows for rapid genotyping of DNA sequence variants without costly post-PCR methods such as direct sequencing [28].

In this study we used HRM analysis to design 42 SNP genotyping assays using whole amplicon melting. Together these assays create a 42-SNP barcode for *P. vivax* that can uniquely identify parasite infections and differentiate their geographic origins, and may ultimately provide insight into changes in parasite population dynamics and transmission.

## Methods

### Bioinformatic identification of candidate barcode SNPs

We identified candidate SNPs for our barcode by performing SNP calling with the Genome Analysis Toolkit (GATK) Unified Genotyper [29] using available sequence data (with quality scores of > 30) on isolates from Central America (Salvador 1 [16]), South America (Brazil I [20] and Acre4 (E. Winzeler, University of California San Diego, personal communication)), Peru (IQ07[30], PQSJ62 (E. Winzeler, University of California San Diego, personal communication), and PQSJ96 (E. Winzeler, University of California San Diego, personal communication) and Asia (North Korea, and India VII) [20]. Additionally, we used available SNP annotation data from sequenced isolates from Cambodia (C08, C15 and C127) and Madagascar (M08 and M19) [19].

### Primer design and assay selection

We designed primer pairs using the LightScanner primer design software version 2.0 (BioFire Defense, U.S.A.). The software uses HRM design parameters including: primer length of 18–28 nucleotides, melting temperature (T_m_) of 58–60°C, GC content of 40–60%, and amplicon size of < 60 base pairs (bp). We then checked the primer pairs for the potential to form primer-dimers or alternative amplicons using Genious software version 6.1 (Biomatters Ltd, New Zealand). If the reaction specificity was acceptable, we screened the primers for species-specificity to *P. vivax* using a BLAST genomic database search (National Library of Medicine (NLM) U.S.A).

### *P. vivax* genomic DNA samples

We used the *P. vivax* strains (North Korea, India VII, and Brazil I) that were sequenced at the Broad Institute [20] as internal controls to identify both the reference and alternate alleles for each assay based on the Sal 1 strain. All three strains are clonal infections that were adapted for growth in monkeys and are publicly available via the Malaria Research and Reference Reagent Resource Center (MR4). The genomic DNA of all three strains was derived from leukocyte depleted monkey blood. Animals were obtained from the National Institutes of Health (NIH)-approved sources, housed in agreement with the Animal Welfare Act and the Guide for the Care and Use of Laboratory Animals (ILAR, 1996); procedures were performed following the NIH Guidelines under protocols approved by the Animal Care and Use Committee of the National Institute of Allergy and Infectious Diseases (Animal Study Protocol LMVR 15).

We used 87 clinical samples for validation of our barcode assays. The samples were derived from blood specimens identified by microscopy to contain *P. vivax* and were collected at study sites in Brazil (31 samples), Sri Lanka (19 samples), Ethiopia (15 samples) and French Guiana (22 samples) with informed consent under human subject guidelines approved by the relevant Institutional Review Boards. The clinical samples from French Guiana were collected by venous blood draw between 2006 and 2010 at study sites located in Cayenne (4), Inland (3), Lower Maroni (4), Upper Maroni (4), Lower Oyapock (4) and Upper Oyapock (3). All 31 clinical samples from Brazil were collected by venous blood draw between 2005 and 2007 in the rural community of Granada [31]. All 19 samples from Sri Lanka were collected between 2003 and 2008 by venous blood draw from patients attending medical health clinics in Trincomalee [32]. All 15 samples from Ethiopia were collected by whole blood spotted on Flinders Technology Associates (FTA) membranes between 2006–2008 from patients with fever attending health clinics in Assendabo [32].

### Ethics statement

For all clinical samples from Brazil the Institutional Review Board (IRB) approval of the study protocol was obtained from the Ethical Review Board of the Institute of Biomedical Sciences of the University of São Paulo, Brazil (773/2007). Written informed consent was obtained from all study participants or their parents/guardians. For all clinical samples from Sri Lanka and Ethiopia the IRB approval was obtained from the Human Subjects Committee of the Harvard School of Public Health (#P10299–106/0209GENE) and the Ethical Review Committee of the Faculty of Medicine, University of Colombo (EC/08/092). Written informed consent was obtained from study participants or their parents/guardians. Illiterate native-speaking study participants who could understand the native language, but were physically unable to talk or write, were entered into the study by oral consent using consent forms that were approved by both the Human Subjects Committee of the Harvard School of Public Health (#P10299–106/0209GENE) and the Ethical Review Committee of the Faculty of Medicine, University of Colombo (EC/08/092). An impartial third party was present to witness the entire consent process and signing of the consent documents. After informed consent was obtained, all samples were de-linked from patient identifiers and provided as discarded samples from the clinic. For all clinical samples from French Guiana the analyzed samples were all obtained by blood collections required by the standard medical care for any patient presenting fever on hospital admission in French Guiana. According to the French legislation (article L.1211–2 and related of the French Public Health Code), biobanking and secondary use for scientific purpose of human clinical remaining samples are possible as long as the corresponding patients are informed and have not given any objection to them. In the present research, this requirement is fulfilled: information is given to every patient through the Hospital brochure entitled ‘‘Information for patients”, and no immediate or delayed patient opposition was reported by the hospital clinicians to the Malaria NRC. Moreover, in application of French legislation (article L.1243–3 and related of the French Public Health Code), samples received at the Malaria NRC had been registered for research purpose in the NRC biobank declared to both the French Ministry for Research and a French Ethics Committee, which both approved and registered this thematic biobank (declaration number DC-2010–1223; collection Nu2). No institutional review board approval is required according to the French legislation.

### DNA sample preparation

Genomic DNA was extracted from clinical samples from using the QIAmp DNA Blood Mini Kit (Qiagen, Germany) [31,32]. We performed whole genome amplification (WGA) on the clinical samples using the Illustra GenomiPhi V2 DNA Amplification Kit (GE Healthcare Bio-Sciences, Piscataway, NJ, USA) according to the manufacturer’s instructions. For each WGA reaction, 1 μl of each clinical sample was used, yielding 40 ul of amplified material. Following WGA, we purified the DNA using Agencourt AMPure XP system (Beckman Coulter, Inc., Beverley, MA, USA) according to manufacturer’s instructions. Following genome amplification, we quantified the concentration of total DNA in clinical samples based on OD_260_ using a NanoDrop 3300 Fluorospectrometer (Thermo Scientific, Waltham, MA, USA). Herein, we refer to the DNA concentration as total DNA, for clinical samples since it contains the presence of both human and *P. vivax* material. All DNA solutions were diluted in 1X Tris-EDTA (TE) Buffer (VWR, Radnor, PA, USA).

### HRM method optimization

We identified the optimal PCR profile in clinical samples as a two-step protocol. The protocol includes: 120 sec at 95°C; 40 cycles of 94°C, 30 sec and 64°C, 60 sec and a final HRM cycle of 95°C, 15 sec; 55°C, 15 sec; and 95°C, 15 sec. The optimized master mix for the PCR reaction contains: 3 μl of DNA sample containing 1 ng/μL DNA in 1X TE Buffer, 1 μL of PCR grade water (VWR, Radnor, PA, USA) 4 μL of 2.5X LightScanner master mix (BioFire Diagnostics Inc., Salt Lake City, Utah, USA), and 2 μL of primer solution containing 0.1 to 0.5 μM of forward and 0.1 to 0.5 μM reverse primers diluted in 1X TE Buffer depending on the individual assay (Integrated DNA Technologies, Inc., Coralville, Iowa, USA) for a total reaction volume of 10 μL. Our studies show that the presence of human DNA did not interfere with the performance of the assays.

To improve the sensitivity as well as the signal-to-noise ratio of the assays in clinical samples, we identified the optimal primer pair concentration for each assay by performing an optimization matrix. We evaluated five different primer concentrations (0.1 μM, 0.2 μM, 0.3 μM, 0.4 μM, and 0.5 μM) and their combinations, leaving all other reaction conditions unchanged. Following amplification, we evaluated the amplicons for the designed product size as described above. Next, we verified genotyping calls for each assay by melt curve analysis using the Eco Real-Time PCR System software version 4.1 (PCRmax, Staffordshire, UK) or the Applied Biosystems ViiA 7 Real-Time PCR System (Life Technologies, Grand Island, NY, USA). We considered a primer concentration to be optimal when the amplification: (1) resulted in an amplicon of the correct size, (2) performed at a cycle threshold (C_t_) less than 30 (as anything greater than 30 could generate unreliable melting profiles [33]), and (3) produced a single peak in the melt profile with no primer-dimer artifacts identified by Eco Real-Time PCR System software version 4.1 or the Applied Biosystems ViiA 7 Real-Time PCR System.

### HRM barcode method

To perform HRM SNP barcoding, we first quantified the concentration of DNA for all clinical samples, as described above using a Nanodrop. We then diluted the samples in 1X TE buffer to a concentration of total DNA (human and parasite DNA) at 1 ng/μl based on based on OD_260_. For all assays, we included sequenced control samples in each assay plate to identify the reference and alternate SNP temperature melt (T_m_) curves. Next, we prepared a master mix (described above) for each assay. We calculated the amount of master mix by multiplying the volume of each component by the number of PCR wells or test samples. We added 7 μl of the master mix to each well in the PCR plate. We gently vortexed and centrifuged the reaction mixture, then added 3 μl of the DNA dilution at 1 ng/μl (final assay concentration 3 ng/μl) to each well for a total reaction volume of 10 μl. We placed a seal on top of the PCR plate with a roller and then centrifuged at 1000 RPM for 1 min. We optimized this protocol to be performed in either a 48-well plate in the Eco Real-Time PCR System or in a 384-well plate in the Applied Biosystems ViiA 7 Real-Time PCR System. We used HRM software on the Eco Real-Time PCR System or the Applied Biosystems ViiA 7 Real-Time PCR System for HRM genotyping analyses. See supplemental materials S1 File for the full barcode assay protocol.

### Genotype determination

To identify a sample genotype we analyzed the derivative T_m_ curve for each assay. Because *P. vivax* is haploid in peripheral blood, we defined the detection of two alleles at any assay position as a polymorphic site and the detection of one allele as a monomorphic site. We considered samples with more than one polymorphic SNP position to be polygenomic. We used the control samples to identify the reference and alternate alleles for each SNP. Because the HRM method relies on saturating DNA dyes, the reference and alternate alleles produce single T_m_ peaks that differ by 0.7–1.2°C [26]. This allowed us to identify monomorphic genotypes by their single T_m_ peak and their alignment with a control T_m_ curve. By contrast, we identified polymorphic genotypes by their skewed or shifted T_m_ curves.

### Genotyping reproducibility

We evaluated assay reproducibility by genotyping samples in duplicate on two different genotyping platforms: the PCRmax Eco (48-well format) and the Applied Biosystems ViiA 7 (384-well format) machines. We screened the 42 assays against three clinical samples and three sequenced controls in duplicate and calculated the difference in T_m_ values for each assay measured on both platforms. We then calculated the mean and standard deviation (SD) of these T_m_ differences to use as a metric of the reproducibility and robustness of each assay on both platforms. Additionally, we evaluated the reproducibility of the 42 assays in our pilot screen by evaluating the variability of the T_m_ value across duplicates for all monomorphic genotype calls from the 87 clinical samples and three control samples.

### Limit of detection and assay efficiency

Following DNA quantification, we evaluated the robustness of the assays by determining the reaction efficiency and limit of detection using the standard curve method. To perform the standard curve reaction, we prepared a 10-fold serial dilution of DNA from both sequenced control and clinical samples at a starting concentration of 300 ng/μl total DNA (human and primate material). We then used these serial dilutions as the template DNA in the qPCR-HRM assays and measured the percent efficiency and identified the minimal DNA concentration. We identified the limit of detection for all assays by setting the criterion of having a cycle threshold (C_t_) of 30, as any C_t_ greater than 30 could generate shifted or unreliable melting profiles [33]. The slope of the standard curve can be translated into an efficiency value and the acceptable standard for assay efficiency is between 90–100% (−3.6 ≥ slope ≥ −3.3).

### Assay detection of mixed genome samples

We evaluated the ability of all assays to detect mixed infections in both the sequenced controls and clinical samples. We quantified the DNA of each sample using the PrimerDesign *P. vivax* probe according to the manufacturer’s protocol and normalized the DNA to 200 copies/μl. We then prepared DNA mixtures that contained the following ratios of reference to alternate allele: 1:10, 1:4, 1:2, 1:1, 10:1, 4:1, and 2:1. Finally, we performed all 42 assays in duplicate using the mixed DNA templates and analyzed the melt profiles to assess the ratios of reference to alternate allele present.

### Evaluation of amplification biases by WGA

We evaluated the ratio of the reference and alternate alleles present in each mixture with and without WGA. First, we identified 10 assays that represented each SNP type in the barcode (CT, CA, GT, and GA) and a pair of monogenomic clinical samples that represented the reference and alternate alleles for each assay. We then prepared mixtures in ratios of 1:1, 1:2, 1:4, 2:1 and 4:1, following the method described above. Since these samples were identified as monogenomic by our SNP barcoding, the resulting mixture had a complexity of infection of two by design. Next, we amplified 1 μl of each mixture and purified the resultant 40 μl (as described in DNA sample preparation). We performed all 10 assays in duplicate and analyzed the melt profiles to assess the percentage of the reference and alternate allele present in each mixture with and without WGA.

### Minor allele frequency (MAF)

We calculated the MAF from allele counts for each SNP in each population. For each polymorphic genotype we counted calls for both the reference and alternate alleles, each with a half contribution compared to monomorphic genotypes. After obtaining the MAF values for each of the populations, we calculated the average MAF (AMAF) as the unweighted mean of the MAF values for the four populations for each SNP. Since we do not know the number of parasite types within a polygenomic sample, the MAF calculation assumes a complexity of infection of two, thus this approach may inflate the MAF calculation.

### Linkage disequilibrium (LD) analysis

We calculated LD using the *r* ^2^ statistic [34]. We calculated *r* ^2^ within each of the four collection sites (Brazil, Sri Lanka, Ethiopia, and French Guiana) for all combinations of SNP pairs that fall on the same chromosome yielding 66 pairs of barcode SNPs. Although the polygenomic samples were included in this analysis, the *r* ^*2*^ calculation was only performed on pairs of genotypes that were monomorphic within such samples. To adjust for background LD induced by small sample size and/or stratification, we computed *r* ^2^ between all pairs of barcode SNPs on distinct chromosomes within each population. For SNPs on the same chromosome, we considered *r* ^*2*^ values significant if they were above a defined percentile in the background levels of LD. This percentile was defined according to a Bonferroni correction at 100*(1–0.05/66).

### Uniqueness within the global sample set

We assessed sample uniqueness by comparing genotypes across all pairs of samples. We counted both pairs of distinct monomorphic genotypes (e.g., A/G) and pairs of monomorphic and polymorphic genotypes (e.g., A/N) as mismatches.

### Population diversity (Barcode π)

To measure population diversity we calculated the ‘barcode π’ statistic, which is the average of number of pairwise differences at assayed SNPs between all members of a population divided by the number of assayed SNPs. In this calculation, we weighted monomorphic/polymorphic genotype pairs at half the value of a monomorphic/monomorphic mismatch. We assessed the variability of barcode π values with 10,000 iterations of nonparametric bootstrapping. Since we do not know the number of parasite types within a polygenomic sample, this calculation assumes a complexity of infection of two, thus this approach may inflate the barcode π values.

### Population divergence (Fixation index: F_ST_)

To measure population divergence we calculated F_ST_ for all pairs of populations. We also generated 100,000 nonparametric bootstrap replicates of 42-SNP barcodes to obtain a 95% confidence interval. For each replicate we calculated the F_ST_ values for each pair for the four populations using the Weir-Hill unbiased estimator [34,35]. To determine whether each of the F_ST_ values represented statistically significant population divergence (i.e., statistical support for rejecting the null hypothesis of a interbreeding (panmictic) population) we performed 100,000 permutations of the population assignments for samples within each pair of populations, then calculated F_ST_. We then performed a Wilcoxon rank-sum test to compare the two distributions of F_ST_ values.

### Principal component analysis (PCA)

We performed PCA with the program SmartPCA in the Eigensoft package [36] on the four populations (Brazil, Sri Lanka, Ethiopia, and French Guiana) together and in pairs. In addition, we performed PCA on the four populations using only the data from a reduced set of SNP assays (either 14 or 28), where we populated the barcode subsets with the assays with the highest AMAF values. We assessed the significance of PCA results using the percent of variance (POV) explained, which is calculated as the sum of the eigenvalues corresponding to the first and second principal components over the sum of all eigenvalues.

### Accession numbers

The genotype data for the studied isolates has been recorded in S1 Table and will be hosted by PlasmoDB [12] (http://plasmodb.org/).

## Results

### Sequence analysis and optimization identifies a 42-SNP barcode set

For the *P. vivax* barcode, we chose SNPs in putatively neutral genomic loci, identified using all *P. vivax* sequence information made available to us through collaborators or community sequencing efforts. Candidate SNPs were restricted to those that are globally polymorphic, having two alleles present in two continental populations—South America and Asia. In these populations, we identified 16,288 SNPs from South America (Brazil and Peru) and 37,721 SNPs from Asia (India and North Korea). Screening these for globally polymorphic SNPs yielded 2,818 candidates. We added an additional 599 SNPs that were polymorphic in Cambodia and in Madagascar *P. vivax* genome sequences. Screening these candidates for those at putatively neutral sites (intergenic, intronic or 4-fold degenerate sites) produced a set of 438 SNPs for HRM assay development.

We winnowed the 438 candidates to a final 42-SNP barcode based on HRM primer design guidelines, species-specificity, and the ability to robustly distinguish the reference and alternate alleles in clinical samples. We designed 187 primers for HRM analysis that were specific to *P. vivax*. We screened these assays for accurate HRM genotyping using *P. vivax* controls. One hundred and fifty-seven of the 187 had a single amplicon of the expected size and accurately called the target SNP as either the reference or alternate allele among sequenced control DNAs. We tested the 157 assays for genotyping accuracy and robustness in a broad panel of clinical samples from Brazil (31 samples), Ethiopia (15 samples), Sri Lanka (19 samples), and French Guiana (22 samples). We identified 42 out of the 157 as high-performing assays (S1 Table) that could successfully distinguish both alleles by HRM with a 0.7–1.2°C T_m_ shift (S2 Table).

### HRM assays are robust and reproducible

We measured the efficiency of the PCR reaction for all assays by performing a standard 10-fold dilution series of the DNA. Using this method, all 42 assays were robust, with a dynamic range in clinical samples of 300 ng/μl DNA with a detection limit of 0.3 ng/μl DNA in 16 out of 42 assays and 0.03 ng/μl DNA in 26 out of 42 assays. The overall efficiency range for all 42 assays was 90% to 100% (S3 Table). We chose 3 ng/μl total DNA as an optimal assay concentration, allowing for a universal PCR method by eliminating the need to rescreen clinical samples with low or poor DNA quantity, which ultimately improves genotyping throughput. By using WGA, we were still able to use a limited quantity of DNA from our *P. vivax* samples. Here, we carried out all assay performance studies and the pilot screen using only 1 μl of each *P. vivax* sample that was amplified by WGA, as described in the methods section.

**Fig 1:**
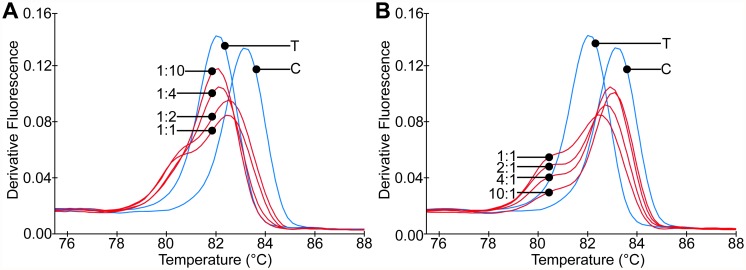
qPCR-HRM assays quantify alleles within DNA mixtures. The derivative melt graphs show the T_m_ profiles of a representative assay (12) tested with mixtures of clinical samples known to contain only one allele at this assay position. The ratios of genomic DNA from samples containing reference (C) allele to alternate (T) allele in the mixtures were: **(A)** 1:10, 1:4, 1:2, 1:1 and **(B)** 10:1, 4:1, 2:1 1:1. The other 41 assays not shown were tested similarly and all performed comparably to detect mixed allelic samples.

We evaluated the sensitivity of all assays to detect polymorphic genotypes by examining the detection limit of both the alternate and reference allele in mixed genome samples in duplicate. All 42 assays were highly sensitive, with a dynamic range in mixed genome samples of *P. vivax* down to a detection limit of 10 copies/μl (S4 Table). The HRM analysis clearly showed the sensitivity of the assays to distinguish each ratio of reference to alternate alleles (1:10, 1:4, 1:2, 1:1, 10:1, 4:1, and 2:1) in mixed genome samples (Fig. 1).

We evaluated assay reproducibility of the 42 assays by screening them against three clinical samples and three sequenced controls. The assays were run in duplicate on two different genotyping platforms: the PCRmax Eco (48-well format) and the Applied Biosystems ViiA 7 (384-well format) Real-Time PCR Systems. All genotype calls (504 out of 504 SNP calls) were concordant across duplicates and on both genotyping platforms. The variability of the T_m_ values for each assay in duplicate was ±0.038°C on the Eco, ±0.046°C on the ViiA 7, and ±0.038°C between platforms (S5 Table). We then compared manual genotyping to the automatic genotyping feature of the ViiA 7. The ViiA 7 genotyping software automatically genotyped 98% (494 out of 504 SNP calls) of the SNP calls accurately. We successfully genotyped the 10 missed calls by manual inspection.

We evaluated the overall reproducibility of the 42 assays in our pilot screen by evaluating the variability of the T_m_ values across duplicates for all monogenomic calls from the 87 clinical samples and control samples. The variability of the T_m_ values across duplicates for monogenomic calls was ±0.070°C on the Eco (S6 Table). The assays were highly sensitive where all 87 samples were successfully genotyped (3654 out of 3654 SNP calls) in duplicate by all 42 assays (S1 Fig).

### WGA does not affect SNP-barcode genotyping accuracy

**Fig 2:**
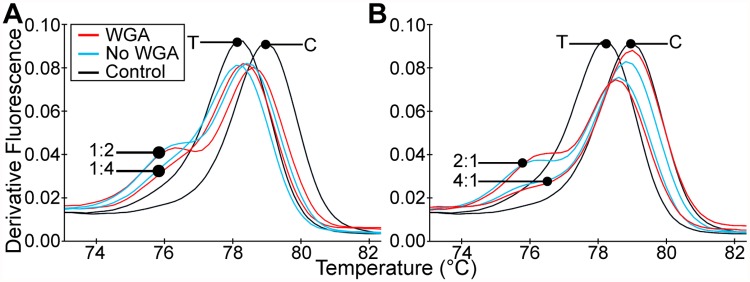
WGA does not affect results in HRM analysis. The derivative melt graphs show the comparison of T_m_ profiles of a representative assay (14) tested with mixtures of monogenomic clinical samples processed with and without WGA prior to PCR. The ratios of reference (T) allele to alternate (C) allele in the mixtures were: **(A)** 1:2 and 1:4 and **(B)** 4:1 and 2:1.

Using a series of known allelic mixtures, we found that WGA prior to PCR did not affect the genotyping accuracy of the HRM assays or resulting genotype. We selected assays representing each SNP type present in the barcode (CT, CA, GT, and GA). We then evaluated mixtures of monogenomic clinical samples with varying ratios of reference and alternate alleles (1:1, 1:2, 1:4, 2:1 and 4:1). The HRM analysis showed that all assay SNP types detected the ratio of reference and alternate alleles with the same accuracy in duplicate in sample mixtures processed with and without WGA (Fig. 2). We further compared the 42 assays using 17 clinical samples from French Guiana with and without WGA, and found no significant differences. For all 42 assays and 17 samples, all genotype calls were concordant between sample treatments.

### 42-SNP barcode captures population diversity

**Fig 3:**
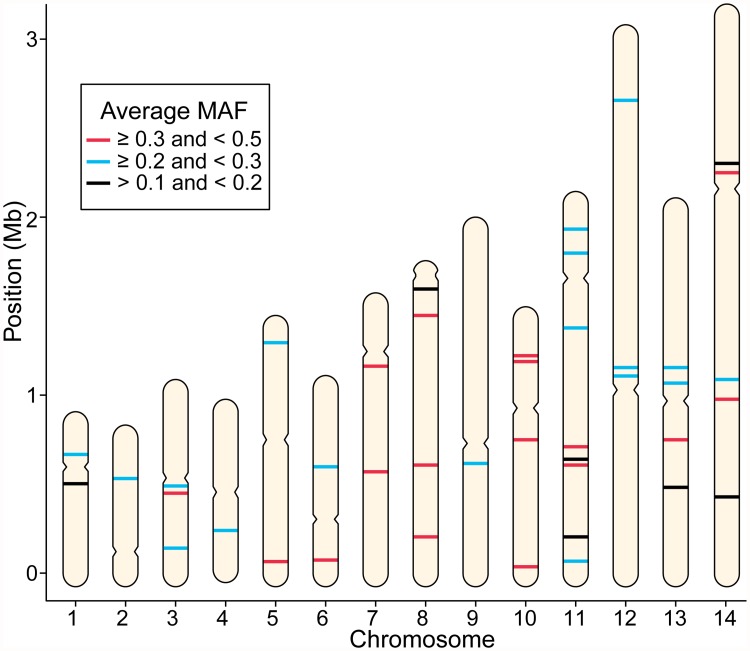
The 42-SNP barcode is distributed across the genome. The location of the 42 SNPs is illustrated on the 14 chromosomes of the *P. vivax* genome. SNPs are colored by their average minor allele frequency (AMAF) among the populations tested; 17 SNPs had AMAF ≥ 0.3, 18 SNPs had 0.3 > AMAF ≥ 0.2, and 7 SNPs had 0.2 > AMAF > 0.1 (S7 Table). Putative centromere locations are indicated by the pinched location on the perimeter of each chromosome [16].

The 42 SNPs span all 14 chromosomes of the *P. vivax* genome, and each SNP is highly informative across four distinct geographic populations: Brazil, Sri Lanka, Ethiopia, and French Guiana. The SNPs captured high degrees of diversity, with the AMAF value for each SNP > 0.1 (Fig. 3, and S7 Table). Moreover, the SNPs were independently informative (S2 Fig). We did not expect SNPs to be in LD since the closest pair of SNPs is 21,237 bp apart (SNPs 5 and 6), which is beyond the map distance over which we typically see significant LD in *Plasmodium* populations [37,38]. Using the *r* ^*2*^ statistic to measure LD (which examines allele correlation from 0 to 1), we found that all SNP pairs had *r* ^*2*^ < 0.53 in each. None of the *r* ^*2*^ values were significantly different from the background LD levels after multiple comparison corrections.

The barcode was able to distinguish all samples except for two pairs of Brazil samples (B13 and B21 and B19 and B20) and one trio of Brazil samples (B1, B4, B8); these samples were identical at all genotypes, including polymorphic loci (S1 Fig). In all populations other than Brazil, each sample pair was separated by at least five differences. Overall, the 42-SNP barcode captured high levels of population diversity, with the median bootstrapping values of barcode π at 0.37 in Brazil, 0.39 in Sri Lanka, 0.36 in Ethiopia, and 0.37 in French Guiana. The 42-SNP barcode also uncovered a high prevalence of polygenomic infections in the global sample set, with 45% in Brazil (14 of 31), 74% in Sri Lanka (14 of 19), 60% in Ethiopia (9 of 15), and 41% in French Guiana (9 of 22).

### 42-SNP barcode detects population divergence

**Fig 4:**
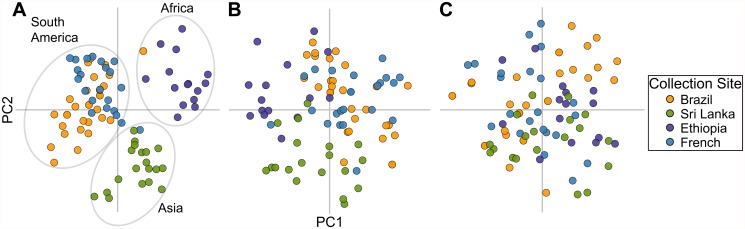
Barcodes with a reduced number of SNPs lose the ability to classify samples by geographic origin. PCA analysis of the global sample set, with samples colored by collection site. **(A)** The 42-SNP barcode shows separation by continent in PCA, and the three continental clusters are circled. Samples from South America appear to show substructure, with Brazil samples dividing into two distinct clusters, one of which overlaps the single cluster of samples from French Guiana. **(B)** A 28-SNP and **(C)** a 14-SNP barcode selected for maximal population diversity were unable to distinguish populations.

While we chose our assays primarily to capture population diversity, seeking SNPs that were highly informative in all populations, we still examined whether the barcode could additionally detect population divergence. With PCA we showed that the 42-SNP barcode visually distinguished populations, separating the 87 samples by collection site with the exception of Brazil and French Guiana (POV = 22) (Fig. 4A). Shorter barcodes of 28 SNPs (POV = 20) and 14 SNPs (POV = 25) selected to maximize the capture of population diversity were unable to distinguish populations (Fig. 4B-C).

**Fig 5:**
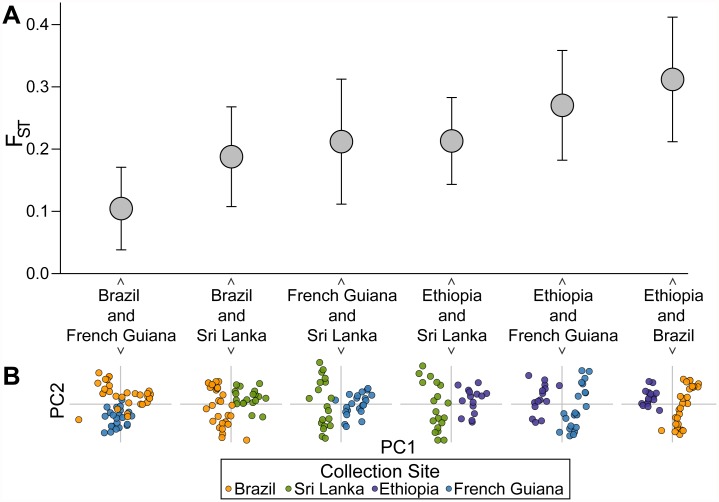
The 42-SNP barcode detects population divergence. **(A)** 95% confidence intervals for F_ST_ values. All F_ST_ values reflect statistically significant population divergence (p < 10^-5^). **(B)** Population pairs separate in PCA with the first two principal components.

We used the median value obtained from bootstrapping of the F_ST_ statistic to quantitatively assess pairwise population divergence, and confirmed the separation visually identified by PCA. We found clear population divergence by both F_ST_ and PCA for Brazil and Sri Lanka (F_ST_ = 0.18; POV = 26), Ethiopia and Sri Lanka (F_ST_ = 0.21; POV = 30), French Guiana and Sri Lanka (F_ST_ = 0.21; POV = 26), Ethiopia and French Guiana (F_ST_ = 0.27; POV = 30), and Ethiopia and Brazil (F_ST_ = 0.31; POV = 33) (Fig. 5A). Bootstrapping analysis rejected the null hypothesis of a panmictic population in each case (p < 10^-5^), showing that all the F_ST_ values capture statistically significant population divergence. Although separation of the samples from the neighboring countries of Brazil and French Guiana was not visually resolved by the first two principal components in PCA (POV = 21), the F_ST_ value was still highly significant (F_ST_ = 0.10, p < 10^-5^) (Fig. 5B).

## Discussion

Here, we present a 42-SNP barcode for *P. vivax* that can identify unique infections, and differentiates parasites from different geographic origins. The barcode is robust and requires only a small quantity of DNA to yield reproducible genotype calls with a limit of detection of 0.3 ng/μl DNA. The 42 SNPs in the barcode are putatively neutral, span all 14 chromosomes of the *P. vivax* genome, and capture unlinked variants with high MAFs in geographically diverse populations. The HRM method is highly sensitive to detection of single nucleotide differences; all SNPs (3654 out of 3654 SNP calls) were successfully genotyped in all 87 *P. vivax* clinical samples tested in our pilot screen.

We chose HRM as our technology because it is a low-cost, rapid genotyping technique that requires minimal training [27,28]. One major advantage of HRM is that it only requires a saturating DNA dye (e.g. LC Green plus) and the corresponding primer sets targeting the SNPs of interest. Thus, the reagent cost per-sample on a per-assay basis is very low at approximately 0.70 USD or 30 USD for the entire 42-SNP barcode. Another advantage is there is no need for post-PCR processing as many real-time PCR instruments are coupled with HRM analysis software creating a rapid genotyping technique where analysis can be completed in 1.5 hours. We have optimized this methodology for universal PCR conditions so that it may be readily applied to multi-well formats (48 or 384). In addition, this method is sensitive and accurate even after WGA processing [39], which may be a necessary first step in genotyping [10] due to the limited amount of DNA typically obtained from field-collected blood samples and the inability to propagate *P. vivax* by *in vitro* culture [3,4].

We demonstrated the potential of the 42-SNP barcode as a genotyping tool by using it in a pilot screen of *P. vivax* clinical samples from South America (Brazil and French Guiana), Africa (Ethiopia), and Asia (Sri Lanka). The 42-SNP barcode captured uniformly high levels of diversity within populations. While we would expect biologically significant variation in diversity to appear in full sequence data, the uniformity of the barcode π values demonstrates that the 42-SNP barcode is sensitive to loci that are highly polymorphic in all populations. Although diversity levels were similar in all four populations, Brazil was the only population where we found pairs of highly similar or identical barcodes. Previous findings have shown that the Brazilian *P. vivax* population may have undergone inbreeding, which may account for this observation [11,40–42].

The 42-SNP barcode provided sensitive detection and characterization of polygenomic infections. We were able to detect polygenomic infections at a high prevalence in all populations. Because the 42-SNP barcode captures a high degree of diversity, parasite strains in complex infections should differ at many loci and thus produce many polymorphic genotypes. Notably, we observed that samples from Sri Lanka had approximately twice the prevalence of polygenomic infections found in samples from Brazil and French Guiana. This observation aligns with microsatellite comparisons of Asian and South American *P. vivax* populations [43]. With the emergence of increasing SNP genotyping data, computational tools such as COIL [44] have become available and will be necessary to measure the complexity of infection (COI) in this biallelic SNP data (http://broadinstitute.org/infect/malaria/coil/).

The 42 SNPs not only capture diversity among individuals within populations, but also divergence between populations. While shorter barcodes selected to capture high diversity could not detect population boundaries, all pairs of populations diverged significantly with the 42-SNP barcode. The pair of populations with the second-lowest divergence was Sri Lanka and Brazil, and this similarity is supported by a prior study that genotyped 85 SNPs on chromosome 8 [45]. The ability of the barcode to distinguish populations is particularly important in the detection of imported *P. vivax* cases resulting from travelers or migrant workers [7]. Understanding where parasite populations cross regional boundaries could inform elimination strategies, and even studies restricted to a single region would benefit from flagging unusual infections.

### Conclusions

The *P. vivax* 42-SNP barcode provides an important baseline universal assay set to distinguish parasite infections and their geographic origins, and may provide insight into changes in parasite population dynamics and transmission. We chose HRM as our technology platform because it is a rapid genotyping technique that requires limited training and has minimal subjective data due to its visualization software. Thus, independent research groups should be able to employ this common set of genotyping assays, and their data and findings should be portable across studies, allowing the results from individual sites to be more accurately compared and contextualized. We have thus made our assays and protocols available to the *P. vivax* community for their implementation in disease elimination and research efforts (http://broadinstitute/org/infect/malaria/pvivax/).

While we present all 42 assays here in our universal set, over time, as the assays are employed by more researchers, some may choose to use shorter regional barcodes or barcodes designed for specific purposes. The barcode may also evolve to include additional variants as more genomic information about *P. vivax* becomes available. In particular, drug resistance SNPs can be incorporated into barcode extensions as markers for these important phenotypes are identified in the future; this would allow simultaneous tracking of population characteristics and the emergence of drug resistance.

## Supporting Information

S1 Fig Barcodes for the 87 clinical samples. The 42-SNP barcode was used to screen a broad panel of clinical samples from Brazil (B; 31 samples), Ethiopia (E; 15 samples), Sri Lanka (S; 19 samples), and French Guiana (F; 22 samples). The resultant barcode is shown for each sample. The top panel shows the assays number its corresponding reference (Ref.) or alternate allele (Alt.) and chromosome (Chr.) position. The reference allele is shown in white, the alternate allele in black and polygenomic genotypes with both alleles are labeled N and is shown in blue. The genotyping success rate for the 87 clinical samples was 100% (3654 out of 3654 SNP calls). The assays were run in duplicate to obtain the genotypes.
(TIF)

S2 Fig The 42 barcode SNPs were independently informative. Each plot shows *r* ^*2*^ over physical distance for each pair of barcode SNPs that fall on the same chromosome for a single population. All SNP pairs had *r* ^*2*^ < 0.53 in each. None of the *r* ^*2*^ values were significantly different from the background LD levels after multiple comparison corrections.
(TIF)

S1 Table Genomic properties of the 42 HRM assays. The positions of the SNPs detected by the 42 HRM assays in the barcode are shown along with the chromosome (Chr.) on which the SNP resides, and the position (coordinate number from PlasmoDB [12.0]) on that chromosome. Genomic information for each SNP type (Synonymous, Intron, or Intergenic) the Gene ID where the indicated SNP is located, and the reference and alternate alleles.
(XLSX)

S2 Table Primer sequences of the 42 HRM assays. The forward and reverse primer sequences are listed with their corresponding assay concentration and T_m_ (°C) for the reference and alternate allele.
(XLSX)

S3 Table Efficiency and limit of detection of the 42 HRM assays. The PCR efficiency and the detection limit of both the alternate and reference allele is listed for each assay in the 42-SNP barcode. All assays were performed in duplicate.
(XLSX)

S4 Table Limit of detection in mock mixed infections of the 42 HRM assays. The limit of detection for both the alternate and reference allele in mixed genome samples is listed each assay in the 42-SNP barcode. All assays were performed in duplicate.
(XLSX)

S5 Table Genotyping reproducibility of the 42 HRM assays across platforms. The 42 assays were screened using three clinical samples and three sequenced controls. The assays were run in duplicate on two different genotyping platforms: the PCRmax Eco (48-well format) and the Applied Biosystems ViiA 7 (384-well format) Real-Time PCR Systems. The mean T_m_ (°C) value and standard deviation (SD) for all monogenomic calls in duplicate is listed. All genotype calls (504 out of 504) were concordant across duplicates and on both genotyping platforms. Note, (*) indicates one clinical sample that was polygenomic and was excluded from the study, CTL refers to control sample, CS refers to clinical samples: B to Brazil I, I to India VII, and NK to the North Korea *P. vivax* strains. The column labeled number of replicates shows the number (including duplicates) in the study as well the corresponding number of clinical samples and controls.
(XLSX)

S6 Table Genotyping reproducibility of the 42 assays using 87 clinical samples. The mean T_m_ (°C) values and standard deviation (SD) is listed for 87 clinical samples (monomorphic calls) and control samples. All assays were performed in duplicate. Note, CTL refers to control sample, CS refers to clinical samples, B to Brazil I, I to India VII, and NK to the North Korea *P. vivax* strain. The column labeled number of replicates shows the number (including duplicates) in the study as well the corresponding number of clinical samples and controls.
(XLSX)

S7 Table The 42-SNP barcode captures population diversity. The 42 SNPs captured high degrees of diversity for the four distinct geographic populations: Brazil, Sri Lanka, Ethiopia, and French Guiana, with the average minor allele frequency (AMAF) value for each SNP > 0.1. The minor allele frequency (MAF) for each assay across the four populations is also listed. All assays were performed in duplicate.
(XLSX)

S1 File Barcode assay protocol. The barcode assay protocol is described in detail: including HRM method, supplies, and primer sequences.
(DOCX)
