# Supplementary figures and images for: Development of a Single Nucleotide Polymorphism Barcode to Genotype Plasmodium vivax Infections
Source: PLoS Negl Trop Dis. 2015 Mar 17;9(3):e0003539. doi: 10.1371/journal.pntd.0003539 (PMC4362761; doi:10.1371/journal.pntd.0003539)

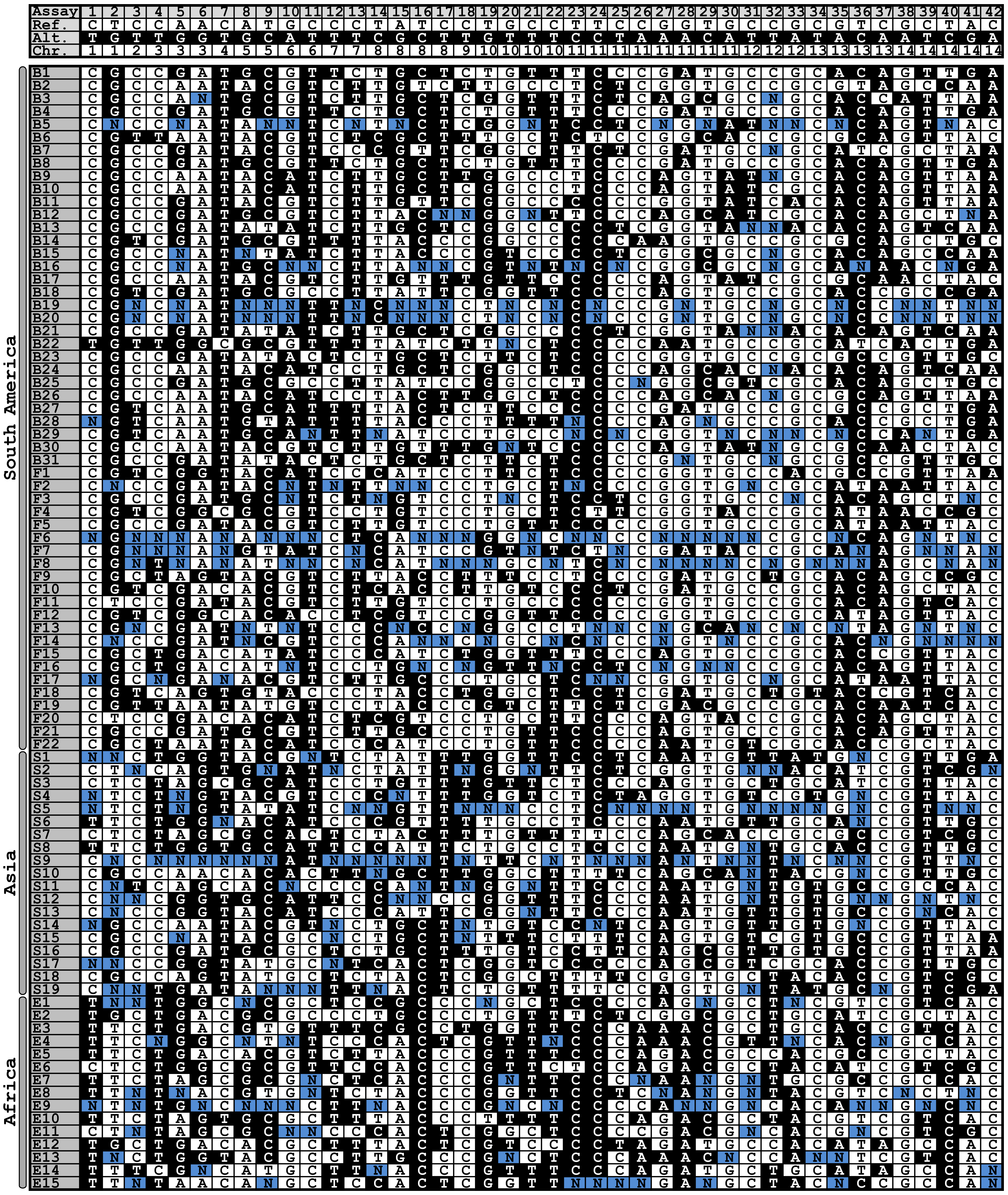

Supplement: S1 Fig — The 42-SNP barcode was used to screen a broad panel of clinical samples from Brazil (B; 31 samples), Ethiopia (E; 15 samples), Sri Lanka (S; 19 samples), and French Guiana (F; 22 samples). The resultant barcode is shown for each sample. The top panel shows the assays number its corresponding reference (Ref.) or alternate allele (Alt.) and chromosome (Chr.) position. The reference allele is shown in white, the alternate allele in black and polygenomic genotypes with both alleles are labeled N and is shown in blue. The genotyping success rate for the 87 clinical samples was 100% (3654 out of 3654 SNP calls). The assays were run in duplicate to obtain the genotypes. (TIF) [file pntd.0003539.s001.tif]

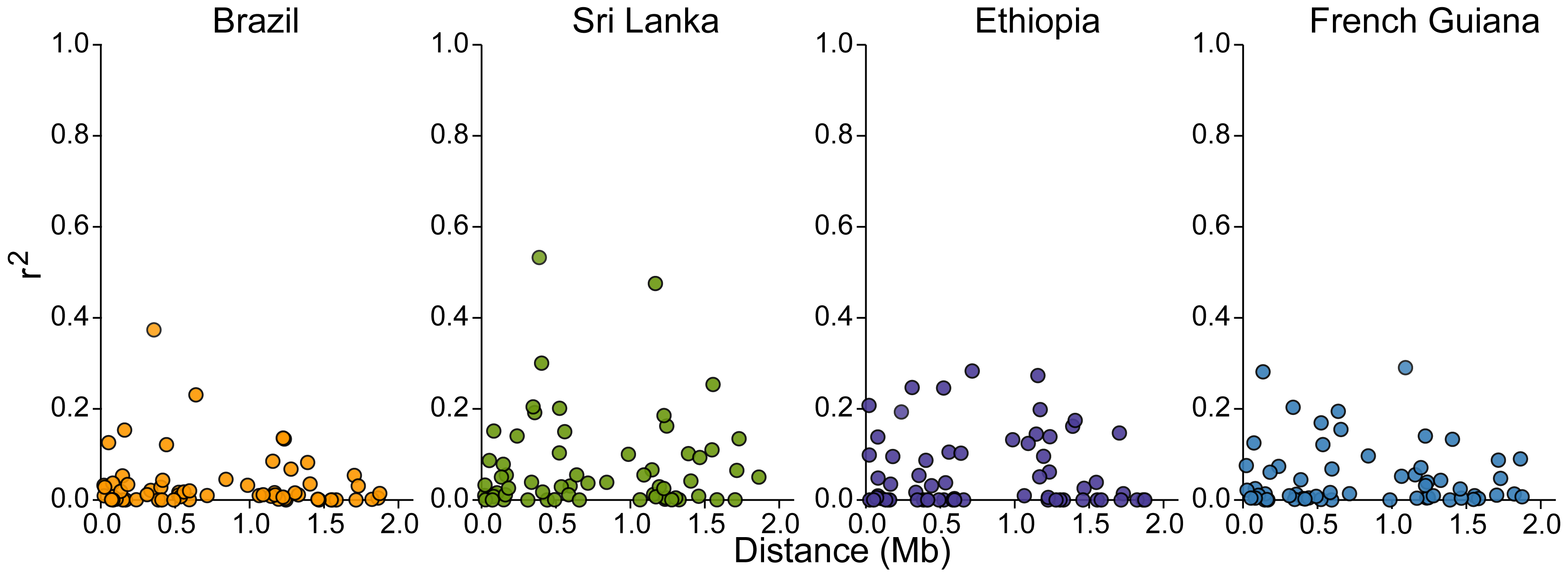

Supplement: S2 Fig — Each plot shows r 2 over physical distance for each pair of barcode SNPs that fall on the same chromosome for a single population. All SNP pairs had r 2 < 0.53 in each. None of the r 2 values were significantly different from the background LD levels after multiple comparison corrections. (TIF) [file pntd.0003539.s002.tif]
