# Supplementary material for: Development of a Single Nucleotide Polymorphism Barcode to Genotype Plasmodium vivax Infections
Source: PLoS Negl Trop Dis. 2015 Mar 17;9(3):e0003539. doi: 10.1371/journal.pntd.0003539 (PMC4362761; doi:10.1371/journal.pntd.0003539)
Supplement: S1 File — The barcode assay protocol is described in detail: including HRM method, supplies, and primer sequences. (DOCX) [file pntd.0003539.s010.docx]

# Protocol: Single Nucleotide Polymorphism genotyping by High Resolution Melt Analysis for *Plasmodium vivax*

This protocol describes the use of high resolution melt (HRM) analysis of 42-Single Nucleotide Polymorphism (SNP) genotyping assays using whole amplicon melting for *Plasmodium vivax* malaria. The SNP identified at each genotyping assay, together creates a 42-SNP barcode for *P. vivax* that will identify individual parasite infections.

**P. vivax 42-SNP Barcode**

# Method

1. **Equipment**

- Pipetters (2 μL, 20 μL, 200 μL, 1000 μL)
- Vortex
- Centrifuge
- HRM instrument

1. **Consumables**

- PCR plates
- Plate seals
- Pipettes
- Eppendorf tubes

1. **Reagents**

- 2.5x Light Scanner Master Mix (BioFire Diagnostics, U.S.A. Catalog # HRLS-ASY-0003)
- Forward and reverse primers (see table for sequences)
- PCR-grade water (supplied in master mix kit)
- Control DNA to identify both the reference and alternate alleles for each assay
- Clinical samples

1. **DNA Sample Preparation**

Quantitate the concentration of total DNA in clinical samples based on OD_260_ using a NanoDrop 3300 Fluorospectrometer (Thermo Scientific, U.S.A.). Alternatively, the genome copy number of *P. vivax* DNA can be quantified in clinical samples by using the Primerdesign *P. vivax* quantitative real-time PCR kit (primerdesign Catalog # Path-P.vivax standard) according to manufacturer’s instructions.

If the amount of DNA sample is limited we recommend performing whole genome amplification (WGA) on the clinical samples using the Illustra GenomiPhi V2 DNA Amplification Kit (Catalog # 25-6600-30) according to the manufacturer's instructions. Following WGA, we purified the DNA using Beckman Coulter Agencourt AMPure XP system (Catalog # A63881) according to manufacturer’s instructions.

1. **Experimental Set-up**

The plate map must include controls for both the reference and alternate allele for each assay. An example of a 48-well and 384-well plate map to test 6 clinical samples in duplicate is shown on page 3.

6. **Setting up the HRM plate and master mix**

| **HRM Master Mix cocktail** | **1X (**μL) |
| --- | --- |
| Primer mix (For each assay: 0.1-0.5 μM of forward and reverse primers)* | 2 |
| PCR-grade water | 1 |
| 2.5x Light Scanner Master Mix | 4 |
| DNA (1 ng/μl) | 3 |
| Total Volume | 10 |
| *See Table S2 |  |

1. Calculate the amount of HRM master mix needed by multiplying each component (shown above for one reaction (1X)) by the number of PCR wells or test samples. Add 1X to account for volume loss.
2. Make primer mix (See Table S2) in 1X TE Buffer. A stock solution of this mixture can be prepared and stored at 4 °C.
3. Prepare a dilution 1 ng/μl of DNA samples and controls in 1X TE Buffer. Note: if background noise in the melting profile is observed the DNA may have contaminants from the extraction method and a serial dilution of the DNA should be performed to identify the optimal concentration of DNA to genotype your clinical sample.
4. Prepare the HRM master mix
5. Vortex and briefly centrifuge
6. Add 7 μL of the master mix to each well of the plate as defined on the plate map
7. Add 3 μL of DNA to each well as defined on the plate map and gently pipet to mix
8. Seal the plate with an optically clear pressure-sensitive adhesive film
9. Centrifuge at 1000 RPM for 1 minute
10. Place plate in HRM machine start program with defined HRM conditions (below)

**HRM Conditions**

| Temperature (°C) | Time (sec) |  |
| --- | --- | --- |
| 95 | 120 | 40 cycles  (2-Step PCR) |
| 94 | 30 |  |
| 64 | 60 |  |
| 95 | 15 | HRM |
| 55 | 15 |  |
| 95 | 15 |  |

1. **Data Analysis**
2. Adjust the pre- and post-melt normalization regions outside of the melting region
3. Define your genotypes as reference and alternate allele
4. Genotype samples by manual or automatic calling of SNP genotypes

**48-well plate map**

**384-well plate map**
